# Impact of a single bout of resistance exercise on serum Klotho in healthy young men

**DOI:** 10.14814/phy2.15087

**Published:** 2021-10-29

**Authors:** Takuma Morishima, Eisuke Ochi

**Affiliations:** ^1^ Faculty of Liberal Arts and Sciences Chukyo University Aichi Japan; ^2^ Faculty of Bioscience and Applied Chemistry Hosei University Tokyo Japan

**Keywords:** endothelial function, endothelin‐1 resistance exercise, flow‐mediated dilation, Klotho

## Abstract

**Background:**

It has been shown that Klotho protects vascular endothelial function. Given that a single bout of resistance‐exercise‐induced hypertensive stimulus causes endothelial dysfunction, we postulated that acute resistance exercise would reduce serum Klotho levels. In this respect, the reduction in serum Klotho levels would be associated with the response of flow‐mediated dilation (FMD). Therefore, the purpose of this study was to investigate the impact of acute resistance exercise on the Klotho response in serum. In addition, we examined the relationship between the serum Klotho and FMD responses following acute resistance exercise.

**Methods:**

Twelve untrained men participated in this study (20.4 ± 0.3 years). Following baseline measurements (blood pressure, blood collection, FMD), subjects performed leg extensions, which consisted of 10 repetitions for five sets at 70% of one‐repetition maximum. After the exercise, measurement of blood pressure, blood collection, and FMD assessment were repeated for 60 min. We analyzed Klotho and endothelin‐1 (ET‐1) concentrations in blood serum.

**Results:**

As expected, the exercise significantly elevated blood pressure and led to decreased FMD (*p* < 0.05). However, Klotho concentrations were significantly increased following exercise (*p* < 0.05). No correlation was observed in Klotho and FMD responses following acute resistance exercise. However, there was a significant positive correlation between Klotho and ET‐1 in response to resistance exercise (*p* < 0.05).

**Conclusion:**

In conclusion, the present study reveals that serum Klotho significantly increased following a single bout of resistance exercise. However, the increase in Klotho may not associate with the acute reduction in endothelial function.

## Abstract 2

Our study reveals that serum Klotho increased following a single bout of resistance exercise. The increase in the Klotho response in serum did not associate with acute reduction in endothelial function following resistance exercise. The increase in the serum Klotho concentration is not responsible for the temporal impairment of endothelial function following a single bout of resistance exercise in healthy young men.

## 1 INTRODUCTION

Klotho is primarily produced by kidney and is an anti‐aging protein (Kuro, 2019; Kurosu et al., 2005). Previous studies in animal model showed that Klotho modulates the generation of reactive oxygen species (Doi et al., 2011; Yamamoto et al., 2005) and anti‐inflammatory response through the signals such as insulin‐like growth factor‐1 (IGF‐1), Transforming Growth Factor‐β1 (TGF‐β), Wnt (Liu et al., 2007). It has been shown that Klotho has the roles in preventing sarcopenia and atherosclerosis (Kuro, 2019). Interestingly, plasma Klotho concentrations decrease with advancing age in healthy humans (Amaro‐Gahete et al., 2020; Yamazaki et al., 2010) and acts as humoral factor with several vascular protective effects (Six et al., 2014). In fact, higher levels of serum Klotho concentration are associated with higher values of endothelial function evaluated by flow‐mediated dilation (FMD) (Keles et al., 2015). In addition, the reduction in serum Klotho is independently related to the signs of vascular dysfunction including such as arterial stiffness and vascular calcification (Kitagawa et al., 2013). Collectively, Klotho is expected to have positive effects on endothelial function, but the limited data have shown a relationship between Klotho and endothelial function in young healthy humans.

Little is known about the effects of resistance exercise on Klotho. Several studies investigated the influence of long‐term training on blood Klotho responses. For example, Matsubara et al. demonstrated that 12‐week of moderate intensity aerobic training increased plasma Klotho concentrations in postmenopausal women (Matsubara et al., 2014). In addition, serum Klotho levels were also enhanced by moderate intensity training but not sprint interval training in healthy, sedentary men (Middelbeek et al., 2021). To understand the chronic training adaptation, it is of paramount importance to focus on the physiological responses following a single bout of exercise. Interestingly, serum Klotho concentrations were increased with a single bout of running exercise in men and women (Santos‐Dias et al., 2017; Tan et al., 2018). By contrast, a single bout of exhaustive exercise in mice causes a significant reduction in serum Klotho (Rao et al., 2019). Therefore, the blood Klotho concentration after a single bout of exercise is a controversial topic. Although these studies have contributed to a better understanding of the influence of aerobic exercise on Klotho’s response, an important consideration is that no prior study examined the impact of resistance exercise on blood Klotho secretion.

Although some studies indicated inconsistent results (Jurva et al., 2006; Varady et al., 2010), we have reported that acute resistance exercise that induces temporal hypertension caused endothelial dysfunction (Morishima et al., 2018; Morishima, Iemitsu et al., 2019; Morishima, Toyoda et al., 2019, Morishima et al., 2020). Indeed, resistance exercise with a smaller increase in blood pressure does not lead to endothelial dysfunction (Morishima et al., 2018, 2020). Accordingly, given that the secreted Klotho is associated with endothelial function, we hypothesized that a single bout of resistance exercise would reduce Klotho's concentration in blood. That is, we postulated that resistance exercise that led acute hypertension would decrease endothelial function, and the reduction in endothelial function would be related to Klotho's response. To test this hypothesis, we performed FMD and serum Klotho measurement before and after a single bout of resistance exercise in the present study. We also measured serum endothelin‐1 (ET‐1) concentration that is a powerful vasoconstriction factor (Yanagisawa et al., 1988). A single bout of resistance exercise increases plasma ET‐1 concentration (Boeno et al., 2019; Okamoto et al., 2008), and the augmentation of ET‐1 levels inhibit Klotho expression (Wang & Sun, 2014). However, the relationship between serum Klotho and ET‐1 responses following a single bout of resistance exercise is still unclear.

## 2 MATERIALS AND METHODS

### 2.1 Subjects

Twelve young, untrained, male subjects (age: 20.4 ± 0.3 years, height: 174.6 ± 2.3 cm, weight: 64.1 ± 2.5 kg, body mass index: 17.5 ± 2.4 kg/m^2^) were recruited in the present study. The subjects were informed about the purpose of this study and the experimental procedure, and all provided written informed consent. The study was approved by the Ethics Committee for Human Experiments at the Sports Research Center at Hosei University in Japan [ID: 2017–003].

The subjects did not participate in any training programs at the beginning of this study. We recruited and confirmed that the subjects were nonsmokers, with no history or symptoms of cardiovascular, pulmonary, metabolic, or neurological disease. No subjects reported taking medications and supplements.

### 2.2 Experimental procedures

The subjects visited the laboratory twice during the experimental period. At first, the subjects were assessed based on their one repetition maximum (1 RM) for leg extension with the use of weight‐stack machines. Before the 1 RM testing, the subjects performed warmup sets with 10 repetitions at 50% and 70% of the predicted 1RM and following stretching of the major muscle groups that were subjected to the exercises. Subjects then chose a weight based on the previous effort that allowed them to perform three repetitions (approximately 80% of predicted 1 RM). After 1 min of resting period, the load was added until subjects were not able to lift the weight (range: 10%–20%). Resting periods (1–5 min) took place between each attempt. The 1RM was determined within three to five attempts.

In the second visit, subjects were asked to eat a light meal at least 2 h prior to arriving to the laboratory. The method (i.e., a light meal before experiment) has been approved by many researchers in previous studies (Morishima et al. 2018; Morishima, Iemitsu, et al., 2019; Morishima, Toyoda, et al., 2019; Restaino et al., 2015, 2016). In addition, subjects were prohibited to take caffeine and alcohol for at least 10 h. They were also not allowed to perform exercise for 24 h prior to the study visit. The study was conducted in a temperature‐controlled room that was maintained at 23°C. Upon arrival to the laboratory, the subjects were placed in a supine position. Subjects were instrumented with an automated sphygmomanometer (Omron Cooperation, Kyoto, Japan) for periodic measurements of systolic and diastolic blood pressure (SBP and DBP) after resting quietly for 10 min. A polyethylene catheter was then inserted in an antecubital vein and a baseline blood sample was collected. All vascular measurements in the brachial artery were performed in the right arm. Brachial artery is the most popular artery for the assessment of FMD. Duplex‐Doppler ultrasound was used to measure the diameter of the brachial artery and blood velocity (Aixplorer, Supersonic Imagine, Aixen‐Provence, France). A 10 MHz linear array transducer was placed over the brachial artery just distal to the brachial fossa. We marked the landmark to measure same point repeatedly. Simultaneous diameter and velocity signals were obtained in a duplex mode at a pulsed frequency of 5 MHz and corrected with an insonation angle of 60°. The brachial artery FMD was assessed as described previously (Boyle et al., 2013; Fairfax et al., 2015). Briefly, a cuff was placed on the lower arm. Baseline hemodynamics was recorded for 2 min, and the cuff was then inflated to a pressure of 220 mmHg for 5 min (MIST‐1000, SARAYA, Osaka, Japan). Continuous diameter and blood velocity measures were recorded for 3 min following cuff deflation. The recordings of all vascular variables were analyzed offline using specialized edge‐detection software (S‐13037 ver. 2.0.1, Takei Kiki Kogyo, Japan).

After the baseline measurement, the subjects maintained the supine position for 45 min, and a second blood sample and blood pressure data were then collected. The repeated baseline measurement was conducted to confirm that the baseline parameters were stable. After the second blood collection and blood pressure assessment, the subjects performed resistance exercise (leg extension). The exercise intensity consisted of 10 repetitions for five sets at 70% of 1RM. The resting period between all sets was 60 s. The intensity during the resistance exercise was adjusted to allow the subjects to complete 10 repetitions in each set (approximately 70% of 1RM for the first set). The subjects were asked to lift and lower the weight within 1 s and 2 s periods, respectively. In addition, the subjects were instructed to breath out and in when they lifted and lowered their weights, respectively. During the resting period between sets, systolic and diastolic blood pressures were measured. Following the resistance exercise, the subjects reassumed the supine position. FMD, blood pressure assessments, and blood collections were then repeated at 10, 30, and 60 min after resistance exercise.

### 2.3 Data analysis

The brachial artery FMD percent change was calculated using the following equation: %FMD = (peak diameter −base diameter)/(base diameter) ×100. The brachial artery FMD delta change from baseline 1 was calculated with the use of the following equation: ΔFMD = bottom value of FMD (at 10‐, 30‐, or 60‐min post‐resistance exercise) − FMD value at baseline 1. Blood lactate was measured with the use of whole‐blood samples (Lactate Pro 2; Arkray Inc., Kyoto, Japan). Serum samples were obtained by centrifugation for 10 min, and were stored at −80°C until analysis. Serum Klotho and ET‐1 concentrations were measured by enzyme‐linked immunosorbent assay. The intra‐assay coefficients of variation were 3.2% and 3.8% for serum Klotho and ET‐1 respectively. The SBP, DBP, serum Klotho, and ET‐1 delta change from baseline 1 was calculated with the use of the following equation: ΔSBP, ΔDBP, ΔKlotho, and ΔET‐1 = peak values of SBP, DBP, serum Klotho, and ET‐1 concentrations −SBP, DBP, serum Klotho, and ET‐1 concentrations at baseline 1.

### 2.4 Statistical analysis

Before the statistical analysis, we confirmed that all data were normally distributed. A one‐way (time) repeated measures analysis of variance (ANOVA) with Tukey's posthoc testing was performed on all dependent variables. Pearson's correlation was used in correlation analysis. Significance was accepted at *p* ≤ 0.05. Data are expressed as means ±standard deviation (SD).

## 3 RESULTS

The results of other hemodynamics parameters in this experiment (i.e., blood flow, shear rate, hyperemic shear rate area under the curve etc.) were reported previously (Morishima et al., 2018). The average workload during resistance exercise was 69.7 ± 4.0 kg. SBP and DBP were significantly elevated during resistance exercise (*p* < 0.01) but returned to baseline levels thereafter. Resistance exercise significantly reduced brachial artery FMD (both absolute and percent change), and statistical significance was observed at 30‐ and 60‐min post‐resistance exercise (*p* < 0.05, Table 1).

**TABLE 1:** Brachial artery hemodynamics before, during and after resistance exercise

			Duration of resistance exercise						
	Baseline	Baseline2	1st set	2nd set	3rd set	4th set	10 min	30 min	60 min
SBP (mmHg)	120 ± 8	121 ± 7	143 ± 12*	148 ± 13*	144 ± 13*	150 ± 10*	136 ± 15*	120 ± 6	120 ± 7
DBP (mmHg)	70 ± 7	68 ± 4	80 ± 6*	73 ± 9*	74 ± 10*	76 ± 18	65 ± 9	69 ± 8	72 ± 6
Absolute FMD (cm)	0.03 ± 0.01						0.02 ± 0.01	0.01 ± 0.01*	0.01 ± 0.01*
%FMD (%)	7.6 ± 2.8						5.6 ± 1.4	4.1 ± 1.8*	4.1 ± 2.7*
Blood lactate (mmol/l)	1.2 ± 0.3	1.3 ± 0.2					9.7 ± 3.7*	3.0 ± 1.1*	2.1 ± 0.5

Mean ± SD.

All baseline and post measurements were collected in the supine position.

Abbreviations: DBP, Diastolic blood pressure; FMD, Flow‐mediated dilation; SBP, Systolic blood pressure.

* *p* < 0.05 vs. Baseline.

**FIGURE 1:**
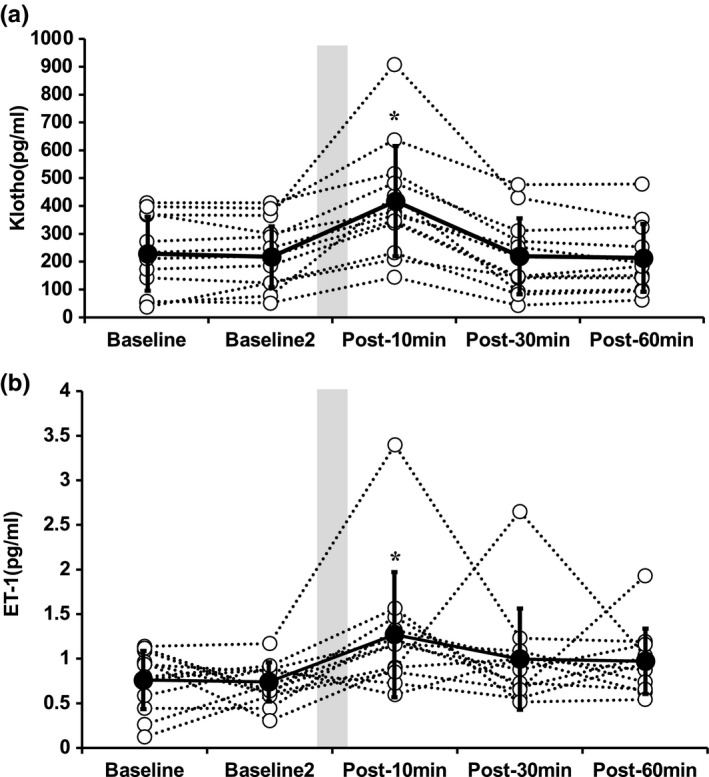
Serum Klotho (A) and endothelin‐1 (B) concentrations before and after a single bout of resistance exercise. Data are expressed as means ±SD. A one‐way (time) repeated measures analysis of variance (ANOVA) with Tukey’s posthoc testing was performed (*p < 0.05 vs. Baseline). Open and closed circles indicate individual and average data, respectively. Shaded box indicates the duration of resistance exercise

Blood lactate levels were significantly elevated in response to resistance exercise (*p* < 0.05). Serum Klotho and ET‐1 concentrations did not change at baselines 1 and 2. However, significant increases were observed in serum Klotho and ET‐1 concentrations at 10‐min post‐resistance exercise compared with baseline 1 (*p* < 0.05, Figure 1). The significances disappeared at 30‐ and 60‐min post‐resistance exercise in both serum Klotho and ET‐1 concentrations.

Baseline serum Klotho did not correlate with any other baseline variables (SBP, DBP, absolute FMD, %FMD, and ET‐1) (Table 2, *p *> 0.05). Similarly, ΔKlotho (peak ‐ baseline) did not correlate with ΔSBP, ΔDBP (peak ‐ baseline), and ΔFMD (bottom ‐ baseline) (Table 3, *p* > 0.05). However, a significant positive correlation was observed between ΔKlotho and ΔET‐1 (peak ‐ baseline) (Table 3, *p* < 0.05).

**TABLE 2:** Correlations between baselinse Klotho concentrations and other variables

	Baseline Klotho (pg/ml)	
	*r*	*p*
Baseline SBP (mmHg)	−0.049	0.879
Baseline DBP (mmHg)	0.144	0.654
Baseline absolute FMD (cm)	−0.114	0.596
Baseline %FMD (%)	−0.011	0.960
Baseline ET−1 (pg/ml)	−0.158	0.462

Abbreviations: DBP, Diastolic blood pressure; ET‐1, Endothelin‐1; FMD, Flow‐mediated dilation; SBP, Systolic blood pressure.

**TABLE 3:** Correlations between Δ Klotho concentrations and other variables

	Δ Klotho (pg/ml)	
	*r*	*p*
Δ SBP (mmHg)	0.130	0.688
Δ DBP (mmHg)	−0.396	0.203
Δ Absolute FMD (cm)	−0.241	0.257
Δ ET−1 (pg/ml)	0.591*	0.002

Abbreviations: Δ, Changes in peak (SBP, DBP, ET‐1, Klotho) and bottom (FMD) values minus baseline; DBP, Diastolic blood pressure; ET‐1, Endothelin‐1; FMD, Flow‐mediated dilation; SBP, Systolic blood pressure.

* Significant correlation with ΔKlotho.

## 4 DISCUSSION

In the present study, we found that the serum Klotho concentration was significantly increased in response to a single bout of resistance exercise. However, the increase in the Klotho response in serum did not associate with acute reduction in FMD following resistance exercise. These data report the novel physiological finding that the increase in the serum Klotho concentration is not responsible for the temporal impairment of endothelial function following a single bout of resistance exercise in healthy young men.

Previous studies have investigated the impact of acute and long‐term aerobic exercise on blood Klotho responses (Baghaiee et al., 2018; Matsubara et al., 2014; Ramez et al., 2019; Rao et al., 2019; Santos‐Dias et al., 2017; Tan et al., 2018). However, no study examined whether a single bout of resistance exercise affects blood Klotho concentration. Conversely, a hypertensive stimulus induced by a single bout of resistance exercise acutely induced endothelial dysfunction (Morishima et al., 2018, Morishima, Iemitsu, et al., 2019; Morishima, Toyoda, et al., 2019; Morishima et al., 2020). Given that Klotho is thought to positively affect endothelial function (Keles et al., 2015), we hypothesized that the serum Klotho concentration would decrease following a single bout of resistance exercise. However, inconsistent with our postulate, Klotho concentration in serum was significantly increased after a single bout of resistance exercise in the present study. Interestingly, exercise training‐induced increase in lean mass index is positively correlated with increases in plasma Klotho levels after a training period (Amaro‐Gahete et al., 2019), thus suggesting that serum Klotho may be related to whole‐body muscle mass. Indeed, grip strength that is a traditional index of whole‐body muscle strength is associated with plasma Klotho concentration in mice (Semba et al., 2012). These observations indicated that blood Klotho would be related to physical fitness as it pertains to muscle mass and strength. The combination of the findings of earlier studies and the present study indicates that the acute increase in serum Klotho concentration after resistance exercise may contribute the augmentation of baseline Klotho levels after long‐term exercise training. Therefore, our work provides significant evidence about the relationship between Klotho and exercise.

To our knowledge, this is the first study used to investigate the relationship between serum Klotho and endothelial function responses following a single bout of resistance exercise. Secreted Klotho diminished cellular apoptosis and senescence, which impair endothelial function in the vascular endothelial cells (Ikushima et al., 2006). Although chronic inflammation and oxidative stress impair endothelial function (Rodriguez‐Manas et al., 2009), Klotho has a counteractive effect to these inflammation (Maekawa et al., 2009) and oxidative stress (Wang et al., 2012). In mice, secreted Klotho from kidney suppressed TGF‐β1 that induces oxidative stress (Doi et al., 2011). Moreover, secreted Klotho participates in the regulation of nitric oxide production in the endothelium (Saito et al., 1998, 2000). As we mentioned above, it has been reported that acute hypertension induced by resistance exercise temporary impaired endothelial function. Based on the literature listed above, we expected that the reduction in FMD after resistance exercise negatively correlated to changes in Klotho responses in serum. In the present study, consistent with previous works, significant reduction in FMD and increases in blood pressure (SBP and DBP) were observed after a single bout of resistance exercise. However, we did not find any correlation between changes in serum Klotho and FMD following acute resistance exercise in the present study. Similarly, no correlation was revealed in serum Klotho response, and SBP and DBP elevations after the resistance exercise, regardless of the fact that a previous study reported that in vivo expression of Klotho in kidney prevents progression of spontaneous hypertension (Wang & Sun, 2009). It is likely that the acute impairment in endothelial function following a single bout of resistance exercise is an independent phenomenon of Klotho response. Although we cannot explain why the increase in serum Klotho concentration induced by muscle contraction (i.e., resistance exercise) do not accomplish the prevention of impairment in endothelial function in response to hypertensive stimulus, the hypertensive stimulus would have a greater effect for endothelial function than Klotho.

ET‐1 is recognized as the most potent endogenous vasoconstrictor (Yanagisawa et al., 1988), and has been indicated to markedly impair endothelial function as assessed by FMD (Nishiyama et al., 2017). Moreover, inhibition of Klotho in brain enhanced ET‐1 secretion (Wang & Sun, 2010). In hypertensive rats, Klotho mRNA and protein levels were lower than those in the normal rats, while ET‐1 mRNA and protein were more than those of normal rats (Tian et al., 2011). The elevation of ET‐1 levels could be reversed in vivo expression Klotho (Wang & Sun, 2014). Therefore, we postulated that serum Klotho and ET‐1 would response in the opposite way. However, changes in serum Klotho concentration in response to resistance exercise were positively correlated to serum ET‐1 concentration in the present study. The reason for the discrepancy in the results is not clear at present because this is the first study to examine serum Klotho and ET‐1 responses after acute resistance exercise in the same subjects. The accumulation of evidence will be needed in the future study.

In conclusion, the present study revealed that serum Klotho concentration was significantly increased following a single bout of resistance exercise. However, the increase in serum Klotho levels did not associate with the acute reduction in endothelial function. Collectively, this work contributes to the better understanding about the Klotho and exercise‐induced cardiovascular response.

## CONFLICT OF INTEREST

No conflicts of interest, financial or otherwise, are declared by the authors.

## AUTHOR CONTRIBUTIONS

E. O. and T. M. conceptualized and designed the study; T. M. performed experiments and analyzed data; E. O. and T. M. interpreted the results of experiments; T. M. prepared Figure and Tables. E. O. and T. M. edited and revised manuscript; E. O. and T. M. approved the final version of article.
